# Three-Dimensional Zeolitic Imidazolate Framework-8 as Sorbent Integrated with Active Capillary Plasma Mass Spectrometry for Rapid Assessment of Low-Level Wine and Grape Quality-Related Volatiles

**DOI:** 10.3390/molecules29246053

**Published:** 2024-12-23

**Authors:** Morphy C. Dumlao, Liang Jiang, Saroj Kumar Bhattacharyya, William A. Donald, Christopher C. Steel, Leigh M. Schmidtke

**Affiliations:** 1 Gulbali Institute, Charles Sturt University, Wagga, NSW 2650, Australia; jlesca@gmail.com (L.J.);; 2 The Australian Research Council Training Centre for Innovative Wine Production, University of Adelaide (Waite Campus), Urrbrae, SA 5064, Australia; 3 School of Chemistry, University of New South Wales, Sydney, NSW 2052, Australia; w.donald@unsw.edu.au; 4 School of Agricultural, Environmental and Veterinary Sciences, Faculty of Science, Charles Sturt University, Wagga, NSW 2650, Australia; 5 Solid Sate & Elemental Analysis, Mark Wainwright Analytical Centre, University of New South Wales, Sydney, NSW 2052, Australia

**Keywords:** zeolitic imidazolate framework-8, active capillary plasma mass spectrometry

## Abstract

The most commonly used methods to chemically assess grape and wine quality with high sensitivity and selectivity require lengthy analysis time and can be resource intensive. Here, we developed a rapid and non-destructive method that would help in grading and decision support. In this work, we demonstrate that integrating a three-dimensional (3D) material for volatile sampling with mass spectrometry detection can be used to sample grapes for phytosanitary, quality or smoke-taint assessments at low levels of marker compounds. An efficient zeolitic imidazolate framework-8 (ZIF-8) material was synthesised in situ on nickel foam (NF), taking advantage of its ultrahigh surface area, structural diversity, and functionality as an emerging nanostructured material for preconcentrating low-level wine and grape quality-related volatiles. When used as a sorbent in thermal desorption tubes and coupled directly to active capillary mass spectrometry, the average signal across the selected analytes increased by ~50% as compared to Tenax TA, a commercially available polymer, in a measurement that takes less than two minutes. The first integration of 3D materials into mass spectrometry opens new possibilities for developing new material architecture with enhanced selectivity of next-generation multifunctional instrumentation for volatile analysis and product quality assessment.

## 1. Introduction

The quality assessment of wine and grapes is commonly evaluated by sensory analysis, which involves measuring differences by analysing and interpreting reactions to products as perceived by sight, smell, and taste [1]. Mostly, this testing leads to unreliable and inconsistent grading due to the subjective nature of such assessment [2]. The most commonly used methods for chemical characterisation with considerable sensitivity and selectivity are mass spectrometry hyphenated techniques, such as gas chromatography–mass spectrometry (GC-MS) and liquid chromatography–mass spectrometry (LC-MS). These methods have been employed for quality assessment, the differentiation of wines (e.g., by origin, variety, and aging), and detecting volatile biomarkers (e.g., smoke taint and fungal exposure) [3,4]. *Botrytis cinerea*, a fungal pathogen of grapes and other horticultural crops responsible for grey mould, produces a variety of volatile off flavours and aromas in wines (e.g., 3-octanone, 1-octen-3-ol) [5,6], and quantification of volatiles associated with the fungus has been used to indicate the severity of infection [7]. *Brettanomyces bruxellensis* is another fungal organism responsible for wine spoilage resulting in a medicinal or barnyard smell, with low molecular weight compounds 4-ethylguaiacol (4EG) and 4-ethylphenol (4-EP) associated with the growth of this organism [8]. Aside from off flavours associated with the growth of fungal organisms, smoke-exposed grapes also pose a significant problem to the wine industry. Volatile phenols from smoke are rapidly taken up by grapes, which adversely affects wine made from these grapes. The presence of volatile phenols and their derivatives, such as guaiacol (2-methoxyphenol), together with their glycoside conjugates, are indicators of smoke-exposed grapes [9,10]. Both 4-ethylphenol and 4-ethyl guaiacol may also be derived from toasted oak barrels during wine maturation and be present at levels that exceed their sensory thresholds of 605 and 110 µg/L in red wine [11]. Reported concentrations for oak-matured wines range from 1–2660 ug/L and 2–437 ug/L for 4-ethylphenol and 4-ethyl guaiacol, respectively [12,13], reflecting highly varied levels of Brettanomyces’ off flavour development. Reported concentrations in Verdelho wines made from grapes purposely exposed to smoke are much lower, at 59 and 128 ug/L [14], although it could be reasonably expected that much higher levels occur in grapes and the resulting wines naturally exposed to intense bushfire smoke. These compounds are attributed to the ‘ashy’, ‘drying’, ‘bitter’, ‘smoked meats’, and ‘dirty’ aftertaste descriptors for wine. Fungal contamination and smoke exposure have significant economic impacts on the global grape and wine industries [15,16].

Some phytosanitary volatiles, including the earthy aroma and mushroom taints produced by fungi, have very low olfactory thresholds in the ng/L range when they colonise grape berry tissues [5,6]. A common method to capture these volatiles directly without damaging the sample (grapes or wine) is either thermal desorption tubes with solid phase materials or long-term exposure of SPME fibre then coupled with GC-MS for detection [17,18].

With emerging nanostructured materials, metal–organic frameworks (MOFs) have been suggested as an alternative sorbent or SPME materials in collecting volatile organic compounds (VOCs) [19,20,21]. These classes of nanosized crystalline materials are characterised by coordination bonds between metal ions and organic molecules. They are known to be versatile for gas storage, adsorption, and separation due to their intriguing structures, tuneable functionality, thermal stability, high surface area, and rich host–guest chemistry [22,23,24]. For example, granular MOF-5 has demonstrated improved sensitivity for formaldehyde detection compared to commercially available porous polymers such as Tenax TA [25,26]. This improvement was attributed to the combined interactions, explained through theoretical modeling, involving the carbonyl group of formaldehyde interacting with the metal sites and the van der Waals interaction between the hydrocarbon “tail” of the carbonyl group and the MOF’s linkers [25]. Results from another study corroborate this observation, showing that the performance of MOF-5 was 53 times and 73 times better than Tenax TA and Carbograph 1 TD, respectively [22]. This enhancement is due to the binding affinity with the zinc–carbon sites of MOF-5, which are absent in both sorbent materials [26]. However, some MOFs are not stable in moist environmental conditions, especially when collecting gas mixtures in the field or involving aqueous extraction. This instability arises primarily from unfavourable conditions that lead to an irreversible hydrolysis reaction with the inert metal clusters within the MOF. If the metal coordination center in the MOF is not sufficiently inert, water can coordinate with the metal cluster, distorting or destroying the MOF’s crystal lattice. Furthermore, the energetics of this interaction must be significant enough to overcome the activation energy barrier of the reaction [27]. ZIF, a subclass of MOF, has a flexible pore aperture and superhydrophobic pore surfaces due to the presence of transition metal ions (e.g., Zn, Fe, Cu, Co) linked with imidazolate forming a 3D tetrahedral framework. Specifically, ZIF-8 has a pore aperture size of approximately 12 Å, along with a narrow aperture of about 3.4 Å. It exhibits electrostatic interactions between the free energy of adsorption and the adsorbate’s dipole moment, which contribute to its molecular sieving effects. Furthermore, the chemical topology of ZIF-8, with a metal–imidazole–metal angle of approximately 145°, is similar to that of zeolites (Si–O–Si angle) and provides a superhydrophobic surface [27,28,29,30]. In addition, ZIF-8 provides a molecular sieve for branched alkanes, aromatics, and heavily halogenated compounds as well as high selectivity with high bio-alcohols recovery, e.g., ethanol and butanol, from its fermentation medium [28,31].

The challenge of high-cost sample preparation, longer turnaround time, complex instrumentation setup, and, more broadly, the high maintenance cost of the equipment, limits the current analytical methods. Integrating new emerging materials such as MOF [32] with current methods (e.g., GC-MS) is not sufficient to address these challenges. Alternatively, dielectric barrier discharge ionisation (DBDI), an ambient mass spectrometry (AMS) technique, is a rapid and direct surface sampling method with little to no sample preparation [33,34,35]. It is known to be less susceptible to ion suppression compared to commercially available plasma-based sources, which can lead to detection enhancement [34]. It is also a softer ionisation, which can lead to easier molecular identification as it typically produces mainly protonated or deprotonated molecules with less precursor ion fragmentation than alternate ion sources such as electrospray ionisation [36,37]. Such characteristics are important in an ambient MS technique due to the complex mass spectra generated of all ionisable compounds in each sample, which are observed simultaneously. Although bypassing sample preparation and chromatography can more readily result in ion suppression effects, AMS ion sources can be easily tailored and designed to suit specific applications such as forensics, clinical, and environmental where rapid screening and monitoring are desirable [38,39].

**Figure 1:**
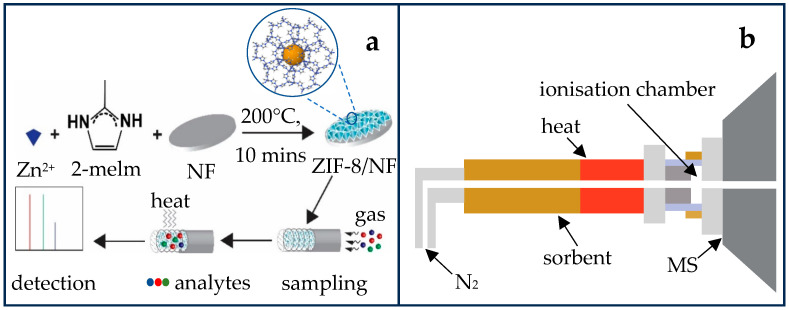
(**a**) Scheme for synthesis, sampling, and detection; (**b**) schematic diagram of coupling the thermal desorption and active capillary plasma mass spectrometry.

In this study, we developed a rapid analytical method by integrating high-efficiency absorbent 3D metal–organic frameworks and active capillary plasma mass spectrometry to detect low-level concentrations of volatiles associated with phytosanitary and quality aspects of grape and wine. The nano-size crystals of ZIF-8 are rapidly and fully grown in situ on NF using a modified solvent-free hot press technique. The 3D ZIF-8/NF is used as a sorbent material held in the thermal desorption (TD) tube. The performance evaluation of the TD tube is done using GC-MS. As a proof-of-concept, selected wine and grape-associated volatiles were rapidly detected by using a TD tube packed with ZIF-8/NF directly coupled to an active capillary plasma ionisation source via a portable thermal desorption system (Figure 1).

## 2. Results and Discussion

For the realisation of rapid detection of low-level phytosanitary volatiles as an important part of agricultural products’ quality assessment, the following general steps were performed: preparation of ZIF-8/NF as a sorbent material, collection of gas samples using the sorbent material (Figure 1a), and rapid detection of analytes using home-built thermal active capillary plasma mass spectrometry (Figure 1b).

### 2.1. Fabrication and Surface Characterisation of ZIF-8 on NF

The ZIF-8 was grown in situ on NF for 10 min using a modified solvent-free hot-press technique [40]. The applied temperature and pressure during the synthesis initiated the rapid formation of nanocrystals ZIF-8 on the NF backbone. Instead of the usual nanocrystals ZIF-8 formation, the resulting reaction formed into highly stable ZIF-8 coatings evident by white crystals [40]. This stable coating disc, when packed into a thermal desorption (TD) tube, could possibly lead to a lower pressure drop and improve mass and heat transfer, which then allows higher gas velocity to capture more target analytes and can lead to a shorter time in the adsorption/desorption cycle [41].

**Figure 2:**
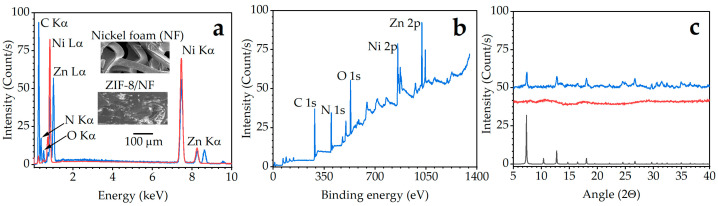
Surface characterisation: (**a**) SEM-EDX profile, (**b**) XPS survey spectrum, and (**c**) XRD pattern; ZIF-8/NF (**blue**), NF (**red**), and reference (**black**).

The morphology, degree of crystallinity, thermal stability, and chemical functionality of the synthesised ZIF-8/NF was evaluated using scanning electron microscopy with an energy dispersive X-ray (SEM-EDX), X-ray photoelectron spectroscopy (XPS), and X-ray diffraction analysis (XRD) (Figure 2). The SEM micrograph of ZIF-8/NF (Figure 2a) shows the ZIF-8 crystals were successfully grown in situ on the NF with particle sizes ranging from ~10 nm to 1 µm [42]. The EDS analysis was carried out to ascertain the presence of C, Ni, Zn, and N (Figure 2a). The observed decrease of Ni intensity on the ZIF-8/NF (Figure 2a, blue) layer suggests high coverage of ZIF-8 on the substrate. The well-distributed ZIF-8 crystals along the NF backbone extended the material’s surface area exposure and, at the same time, may prevent a high-pressure vacuum, thereby optimising its adsorption capability in preconcentrating low-level volatile analytes.

The newly formed ZIF-8/NF was also characterised by XPS. The binding energy of Zn 2p, O 1s, N 1s, C 1s, and Ni 2p (Figure 2b), together with their individual region at higher resolution (Figure S1a–f), were monitored. All expected ZIF-8 features, specifically Zn (coordinating metal), N, and C (imidazole linker) together with the Ni substrate, were consistent with those previously reported in the literature [43,44]. The binding energy peaks located at 530.9 eV, 855.3 eV, and 1021.5 eV indicate the presence of O 1s, Ni 2p, and Zn 2p, respectively. The binding energy values of 284.8 eV and 285.8 eV scanned at higher resolution correspond to C–sp3 and C–N bonds imidazolate ligands, respectively (Figure S2d). The peak at 398.8 eV of the XPS spectrum of N 1s confirms the presence of a C–N–C bond (pyrrole-like nitrogen) from ZIF-8 imidazole groups (Figure S2f).

The individual XRD patterns of ZIF-8/NF (**blue**), NF (**red**), and the reference (**black**) are shown in Figure 2c. The identical patterns confirmed the successful synthesis of ZIF-8 on NF. The characteristic diffraction peaks of ZIF-8 at 2θ  =  7.4°, 10.4°, 12.7°, 18.0°, 22.1°, 24.5°, 26.7°, and 29.6°, which corresponded to planes of (011), (002), (112), (222), (114), (233), (134), and (044), respectively, can be clearly seen [45]. However, the peaks at 14.7° and 16.4°, which correspond to planes (022) and (013), were not clearly visible. The prominent peaks agreed well with previous reports, confirming the typical sodalite structure of ZIF-8 [45].

### 2.2. Loading and Temperature Dependent Study of ZIF-8/NF

Based on the synthesis method, ZIF-8/NF was synthesised using different weights of starting material (50%, 100%, and 150%) with identical molar ratios with the aim of increasing the material loading (Figure S2). The peak intensity in the XRD patterns showed a significant increase as the weight of the starting material increased. This visible increase suggests higher coverage of ZIF-8 on NF. This observation is supported by an earlier study showing the loading increase from 25% to 150% [40]. However, some of the peaks in the XRD pattern at 100% are observed to be less intense compared to those in the XRD pattern at 50%. This could be attributed to variations in the loading technique we applied, which might have contributed to the preferred orientation of the crystal planes, thereby affecting the observed intensity.

Furthermore, the ZIF-8/NF sorbent was subjected to a thermal stability test by using non-ambient XRD experiments. The XRD patterns at increasing temperatures (Figure S3) as well as at 280 °C with seven (7) completed temperature cycles (Figure S4) indicated the thermal stability of the material. This observation is consistent with the previous study showing thermal stability from 200 °C up to 500 °C in N_2_ gas [46,47].

### 2.3. Optimisation of ZIF-8/NF as Sorbent Material via Thermal Desorption Coupled to Gas Chromatography–Mass Spectrometry (TD-GC/MS)

The fully characterised ZIF-8/NF and the newly purchased Tenax TA (Markes International, Bridgend, UK) were the two sorbent materials used in this TD-GC/MS optimisation experiment. The prepared and conditioned TD tubes, as described in Section 3.3—Materials and Methods, were used to collect the three analytes (1-octen-3-ol, 1-octen-3-one, and 3-octanone, *Botrytis* infection indicators). Their analytical performances were then compared using TD-GC/MS.

**Figure 3:**
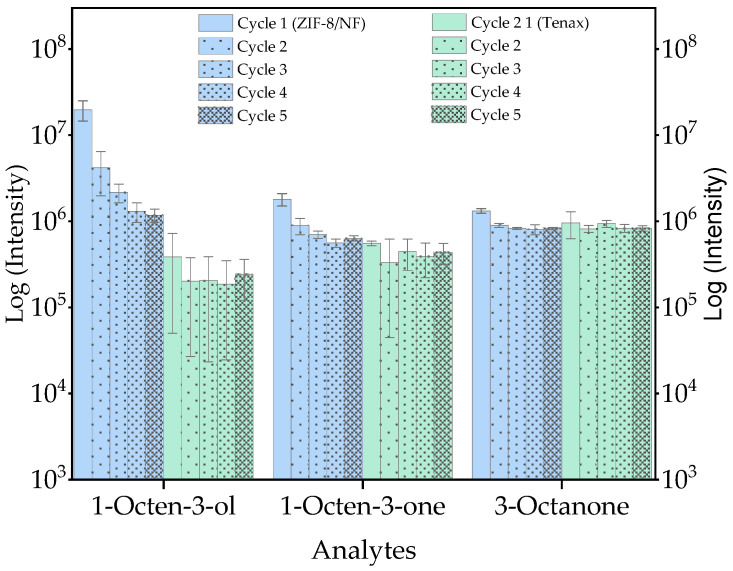
Number of desorption cycles using ZIF-8/NF (**blue**) and Tenax TA (**green**) with a TDU-GC-MS technique.

For this optimisation experiment, five cycles of triplicate runs were done. The log intensity of three analytes, as shown in Figure 3, presents a relatively consistent result across the five cycles in both sorbent materials. The first three cycles of ZIF-8/NF show a slightly diminishing sensitivity for 1-octen-3-ol and 1-octene-3-one, which stabilised thereafter and likely indicates that three conditioning cycles are required for stable analyte detection. Across the cycles, the stability of the material was observed, which corroborates with the experimental results of non-ambient XRD mentioned above (Figures S3 and S4). By TD-GC/MS, the quantification of the selected analytes (1-octen-3-ol, 1-octen-3-one, and 3-octanone) was achieved using mixed standard solutions. Table S1 provides the calibration range, regression coefficient (R^2^), limits of detection (LOD), and quantification (LOQ). It shows that ZIF-8/NF has almost double the LOD of Tenax TA in detecting 1-octen-3-ol, while 1-octen-3-one and 3-octanone have similar LODs for both sorbent materials. It is also observed that 3-octanone using ZIF-8/NF and 1-octen-3-ol and 1-octen-3-one using Tenax TA have greater line gradients, which may infer a higher level of sensitivity. The overall regression coefficient for all the selected compounds was close to one (R^2^ > 0.98), reflecting the linear ranges of the calibration analytical range.

### 2.4. Direct Thermal Desorption Active Capillary Plasma Mass Spectrometry (TD-LTP-MS)

The representative extracted chromatograms of (E)-2-Hexenal, 1-octen-3-one, 3-octanone, guaiacol, and 4-EG using ZIF-8/NF with a thermal desorption temperature at 280 °C are shown in Figure S5. It illustrates that all target analytes elute at less than 1 min, although (E)-2-Hexenal exhibits a very low detection signal. Lower MS signals for (E)-2-Hexenal may arise from its rapid desorption (while the tube is connected to MS) due to the high temperature applied. A desorption temperature-dependent study was conducted to capture all possible analytes of interest. The general observations (Figure S6) indicate that the optimum desorption temperature for all analytes is approximately 200 °C. For (E)-2-Hexenal, guaiacol, and 4-EG, consistent detection is observed across a range of desorption temperatures. This finding contrasts with the expected observation that analytes with low vapor pressure (Table S2) require lower desorption temperatures to be detected. 1-octen-3-one and 3-octanone, which are ketones, demonstrate fairly good detection levels. However, as the desorption temperature exceeds 200 °C, the detection trend between the two ketones shifts favorably toward 1-octen-3-one, likely due to its higher vapor pressure. Nonetheless, vapor pressure may not be the sole factor influencing detection; the interaction between the analytes and the sorbent material also plays a significant role. The surface chemistry interactions between the analytes and the sorbent material using this method warrant further exploration [29,31].

**Figure 4:**
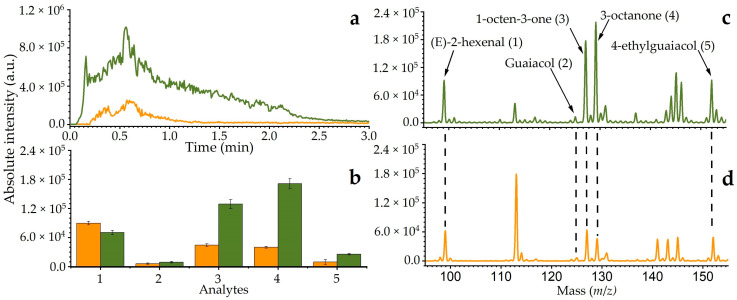
Direct thermal desorption plasma ionisation mass spectrometry using different materials, ZIF-8/NF (**green**) and Tenax TA (**orange**): (**a**) ion chromatogram, (**b**) bar graph of different analytes detected, (**c**) MS spectrum of ZIF-8/NF, and (**d**) MS spectrum of Tenax TA.

In Figure 4a, the chromatogram profiles using sorbents, ZIF-8/NF (**green**) and Tenax TA (**orange**), show that the elution time of the target analytes was reduced to less than 2 min, compared to approximately 30 min for normal GC-MS or LC-MS, indicating a significant reduction in instrumentation time. The integrated area of each chromatogram, ZIF-8 (Figure 4b) and Tenax TA (Figure 4c), shows the detection of various analytes. The detection levels of different analytes for each sorbent, tested in triplicate, are presented in Figure 4d. With ZIF-8/NF, as compared with Tenax TA sorbent, the results indicate that the analyte peak intensities of 1-octen-3-one, 3-octanone, and 4-EG increased by about 70%, while guaiacol showed an increase of 32.4%. In contrast, (E)-2-Hexenal decreased by 27.4%. This observation is plausible due to the interaction of the analyte and the sorbent material, as well as the desorption temperature, as mentioned above, suggesting that ZIF-8 enhanced the detection performance as compared to Tenax TA. The peaks at *m*/*z* 114 and clusters of peaks around *m*/*z* 140 were observed in both sorbents used. Since methanol was utilised as the solvent in sample preparation, these peaks could possibly be attributed to methanol and water cluster formation during active capillary plasma ionisation [48].

## 3. Materials and Methods

### 3.1. Synthesis of ZIF-8 on NF

The ZIF-8/NF was synthesised (Figure 1a) as reported in previous work with modification [40]. Briefly, NF (thickness 1.6 mm, bulk density 0.45 g cm^−3^, porosity 95%, from Good Fellow, Huntingdon, UK) was cut into 0.4 mm discs (diameter). The discs were cleaned by sequential sonication with 3 M HCl, followed by ethanol and water for 30 min, and then air-dried. Zinc nitrate hexahydrate (0.20 g, 1.09 mmol) and 2-methylimidazole (0.50 g, 6.10 mmol) were manually ground and mixed using mortar and pestle. The cleaned NF discs (32 pcs, 0.4 mm diameter) were added and mixed with the ZIF-8 mixture to coat them uniformly. The coated discs were wrapped with aluminium foil, heated at 200 °C for 10 min, and cooled down at ambient temperature before peeling off the aluminium foil. The formed ZIF-8/NF discs were washed with ethanol, dimethylformamide, and water for 1 h each and, finally, stored in ethanol. The ZIF-8/NF discs were dried at 80 °C for 30 min prior to use for material characterisation and thermal desorption (TD) tube preparation.

### 3.2. Surface Characterisation of ZIF-8/NF

Samples for SEM were mounted on a stub with adhesive, and the imaging was carried out using a Hitachi (model: S-3400N, Tokyo, Japan) with a tungsten-filament scanning electron microscope. The surface elemental detection was conducted using Bruker X-Flash 5010, and the data analysis was performed using Bruker Esprit 2.1. X-ray photoelectron spectra were collected on an ESCALAB250Xi (Thermo Scientific, Cambridge, UK) with an Al monochromatic anode at an energy of 1486.68 eV. The survey spectra were collected over the energy range of 0–1400 eV. The high-resolution spectra for each element (Zn 2p, Ni 2p, O 1s, N 1s, and C 1s) were collected over a range of 20 eV at 0.1 eV per step with a pass energy of 50 eV. Data analysis was performed using Thermo Scientific™ Avantage software. All peak positions were calibrated based on the binding energy of the adventitious C 1s at 284.6 eV. The crystal phases of ZIF-8/NF were identified by XRD using a PANalytical Xpert Multipurpose X-ray Diffraction System (MPD) (Malvern Panalytical, Chipping Norton, Australia) with Cu Kα radiation and scanning from 5 to 100°. In situ variable temperature PXRD patterns (Malvern Panalytical, Australia) using a Cu Kα source under N_2_ gas with a 30 min cooling time in between temperature scans from 30 to 300 °C and at least five temperature cycles at 280 °C were taken.

### 3.3. Sorbent Tubes Preparation and Sampling

Two sorbent tubes used were (i) ZIF-8/NF, an in-house prepared material, and (ii) Tenax TA (Markes International, Bridgend, UK). The ZIF-8/NF sorbent was packed using a pre-treated empty thermal desorption (TD) tube (O.D. × L 1/4 in. × 3 ½, from LECO Australia Pty Ltd., Castle Hill, NSW, Australia). The ZIF-8/NF material had an average weight of approximately 90 micrograms (ZIF-8 alone) or 150 g (including the NF), a comparable weight to the Tenax TA. Each tube was packed in the following sequence: stainless steel gauze disc, ZIF-8/NF discs, glass wool, and stainless-steel G-Clip.

Prior to sampling, the ZIF-8/NF and Tenax TA sorbent tubes were conditioned by heating in the tube conditioner system, TC-20 (Markes International, UK), under N_2_ at a flow rate of 40 mL min^−1^. Tenax TA sorbent tubes were conditioned according to the manufacturer’s instructions, with heating the desorption tube at a temperature program of 1° per second until reaching 350 °C, and maintained at this temperature for 2 h. The ZIF-8/NF sorbent tubes were conditioned at 300 °C for 2 h in five consecutive cycles. All the conditioned tubes were tightly sealed and ready for use. For subsequent use, the tubes were conditioned at 280 °C and 320 °C for 30, ZIF-8/NF and Tenax TA, respectively. All the tubes were tested for any contamination using thermal desorption gas chromatography–mass spectrometry as described below.

An individual stock solution of 1 mM in methanol was prepared from the following analytes: (E)-2-Hexenal, guaiacol, 1-octen-3-one, 3-octanone, and 4-EG. Different mixed solutions of varied concentrations were prepared from the stock solution as needed. All solutions were kept at 4 °C. The gas sample was prepared as described previously [49]. Briefly, a 50 µL of methanolic solution containing 100 μM of mixed solution was injected into the pre-heated customised sample setup (Figure S7) using an airtight syringe. The volatiles were collected into nalophan bags for 10 min (approx. 30 L sample volume) and sub-sampled onto triplicates of ZIF-8/NF and Tenax TA sorbent tubes for 10 min (approx. 1 L sample volume) using a calibrated air sampling pump (SKC Inc., Eighty-Four, PA, USA) at a constant flow rate of 100 mL min^−1^. Each TD tube was immediately capped using stainless steel blank nuts fitted with PTFP ferrules. The sampled sorbent tubes were analysed immediately after sampling or wrapped in aluminium foil and stored at 4 °C until analysis. Blank samples were performed to check the background by using pure methanol and analysed with the same procedure.

### 3.4. Thermal Desorption Gas Chromatography–Mass Spectrometry (TD-GC/MS)

The sampled sorbent tubes were analysed using a standard thermal desorption unit, Unity (Markes International, UK), coupled to gas chromatography–mass spectrometry (Agilent 7890N GC, 5975 MSD Agilent Technologies, Santa Clara, CA, USA). The sorbent tubes were thermally desorbed into the GC column using a thermal desorption (TD) unit at 280 °C for 8 min under the flow of helium gas (50 mL min^−1^). The capillary column used for the separation was DB-VRX (30 m × 0.25 mm × 1.4 µm), a low polarity stationary phase column, and helium was used as the carrier gas at a flow rate of 1.6 mL min^−1^. The initial temperature for the GC was set at 50 °C for 2 min, then gradually increased to 220 °C at a heating rate of 15 °C min^−1^ while the *m*/*z* interval ranged from 35 to 335 amu. Chemical compounds were identified by using NIST17 with 85% minimum forward matching quality. The library database was installed into a ChemStation database (MSD ChemStation, Agilent Technologies, USA). The chemical concentration was quantitatively determined by using response factors obtained from a concentration range of 0.5–10 ng/L using the prepared standard gas samples. The method detection limit (MDL) and limit of quantification (LOQ) were determined.

### 3.5. Direct Thermal Desorption Active Capillary Plasma Mass Spectrometry

A custom thermal desorption active capillary plasma mass spectrometry was designed and fabricated, as shown in Figure 1b. The thermal desorption (TD) system was fabricated to fit the 6.35 mm TD tubes with a digital temperature controller. To prepare the TD tube for coupling with active capillary plasma MS, both ends of the TD tube were uncapped, then one end was connected to the N_2_ gas, and the other end was screwed with customised stainless steel (SS) capillary. The N_2_ gas was connected to a digital airflow control system and set to 1.8 L min^−1^ flow to compensate for the vacuum speed of the MS system. Before the start of the analysis, the active capillary plasma source was turned on, and the MS system was activated in running mode. Data were then collected. Then, the TD tube was fitted on the pre-heated thermal desorption system while the SS capillary was positioned carefully into the plasma source before the N_2_ gas was quickly turned on. **Note:** The thermal desorption system is hot, which may cause a burn. Beware of high voltage.

The active capillary plasma ionisation source apparatus was coupled to the portable TD system as described previously, with minor modifications [50]. Briefly, the source was modularly redesigned and fabricated to fit the SS capillary, as previously mentioned above, and the MS inlet. A linear quadrupole ion trap mass spectrometer (Thermo LTQ XL) equipped with a reduced pressure ion funnel (~5 mTorr; Heartland Mobility, Kansas, MO, USA) was used for this study.

The active capillary plasma source was power-driven with square-wave high frequency and high voltage [51]. It consisted of a quartz glass capillary (i.d. 1.5 mm, o.d. 1.8 mm, ca. 2.5 cm) surrounded by an outer Cu electrode (i.d. 1.9 mm, o.d. 2.5 mm, 1.5 cm). The inner electrode, with a conductive ferrule made from a disposable blunt syringe needle (Terumo^®^ from Livingstone, Sydney, Australia; 18 Gauge × 38 mm), was inserted on the axis into the glass capillary and served as the sample inlet. It was designed to improve direct sample injection while generating stable plasma and to facilitate safety and quick replacement or cleaning without turning off or restarting the power.

In this experiment, direct sampling was conducted by direct infusion of 10 µL of 100 µM in the methanolic solution containing at least five (5) target analytes, approximately 125 ng per analyte per tube, into the pre-heated sample set up (Figure S7) using an airtight syringe and purge with ~160 L of high purity N_2_ gas (80 L min^−1^ for 2 min). The volatiles were collected directly into separate sorbent tubes for analysis in triplicate. Two types of adsorbent materials, (i) ZIF-8/NF, an in-house prepared material and (ii) Tenax TA (Markes International, UK), were conditioned, sampled, and tested to evaluate their analytical performance.

## 4. Conclusions

In summary, we successfully synthesised and fully characterised ZIF-8/NF. The one-pot synthesis of ZIF-8/NF is a straightforward, approximately 10 min process that utilises inexpensive and readily available starting materials. According to the literature, ZIF-8/NF is more thermally stable than the polymer material Tenax TA, supporting our findings in loading and temperature-dependent studies.

Using TD/GC-MS, the ZIF-8/NF material demonstrated a twofold improvement in the limit of detection (LOD) for 1-octen-3-ol, while 1-octen-3-one and 3-octanone showed similar LODs compared to Tenax TA. Furthermore, ZIF-8/NF was used for the first time as a sorbent material coupled with active capillary plasma mass spectrometry to capture and detect low-level phytosanitary volatiles. Direct MS results significantly reduced instrumentation time to 2 min for detecting the target analytes, with an approximate 50% increase in detection sensitivity across the selected analytes, and demonstrated a proof of concept for the rapid analysis of volatiles for quality assessment.

This rapid method, with considerable sensitivity, has the potential to support objective quality grading and decision-making in the grape and wine industries. The quick and scalable synthesis of ZIF-8/NF could reduce production costs, and its enhanced performance may enable rapid detection of volatiles in the field, testing the robustness of this proof-of-concept integration of material and mass spectrometry. The newly designed instrumentation could also be automated to facilitate high throughput. Further research will focus on trapping these volatiles on a substrate to allow more robust thermal desorption with active capillary plasma mass spectrometry.

## Data Availability

The data that support the findings of this study are available in the Supporting Information of this article.

## Supplementary Materials

The following supporting information can be downloaded at https://www.mdpi.com/article/10.3390/molecules29246053/s1, Figure S1: Surface elemental binding energies and distributions; Figure S2: XRD profiles of ZIF-8/NF using different weight percent with identical mole ratio; Figure S3: Temperature-dependent study of ZIF-8/NF using non-ambient XRD technique; Figure S4: Non-ambient XRD profile of ZIF-8/NF at 280 °C for seven repeated cycles; Figure S5: The extracted chromatogram at 280 °C desorption temperature using ZIF-8/NF; Figure S6: Desorption temperature dependent study of selected analytes using ZIF-8/NF; Figure S7: Schematic diagram for gas sampling; Table S1: The calibration using TD-GC/MS; Table S2: List of common phytosanitary volatiles in grapes and wines.
