# Supplementary material for: Three-Dimensional Zeolitic Imidazolate Framework-8 as Sorbent Integrated with Active Capillary Plasma Mass Spectrometry for Rapid Assessment of Low-Level Wine and Grape Quality-Related Volatiles
Source: Molecules. 2024 Dec 23;29(24):6053. doi: 10.3390/molecules29246053 (PMC11676309; doi:10.3390/molecules29246053)
Supplement: Supplementary file 1 [file molecules-29-06053-s001.zip › molecules-3361828-supplementary.pdf]

# Supporting Information

# Three-Dimensional Zeolitic Imidazolate Framework-8 as Sorbent Integrated with Active Capillary Plasma Mass Spectrometry for Rapid Assessment of Low-Level Wine and Grape Quality-Related Volatiles

**Morphy C. Dumlao [1,2,3,*], Liang Jiang [1,2,4], Saroj Kumar Bhattacharyya [5], William A. Donald [3], Christopher C. Steel [1,4] and Leigh M. Schmidtke [1,2,4,*]**

[1] Gulbali Institute, Charles Sturt University, Wagga, NSW 2650, Australia; jlesca@gmail.com (L.J.)
[2] The Australian Research Council Training Centre for Innovative Wine Production, University of Adelaide (Waite Campus), Urrbrae, SA 5064, Australia
[3] School of Chemistry, University of New South Wales, Sydney, NSW 2052, Australia; w.donald@unsw.edu.au
[4] School of Agricultural, Environmental and Veterinary Sciences, Faculty of Science, Charles Sturt University, Wagga, NSW 2650, Australia
[5] Solid Sate & Elemental Analysis, Mark Wainwright Analytical Centre, University of New South Wales, Sydney, NSW 2052, Australia
* Correspondence: m.dumlao@unsw.edu.au (M.C.D.); lschmidtke@csu.edu.au (L.M.S.)

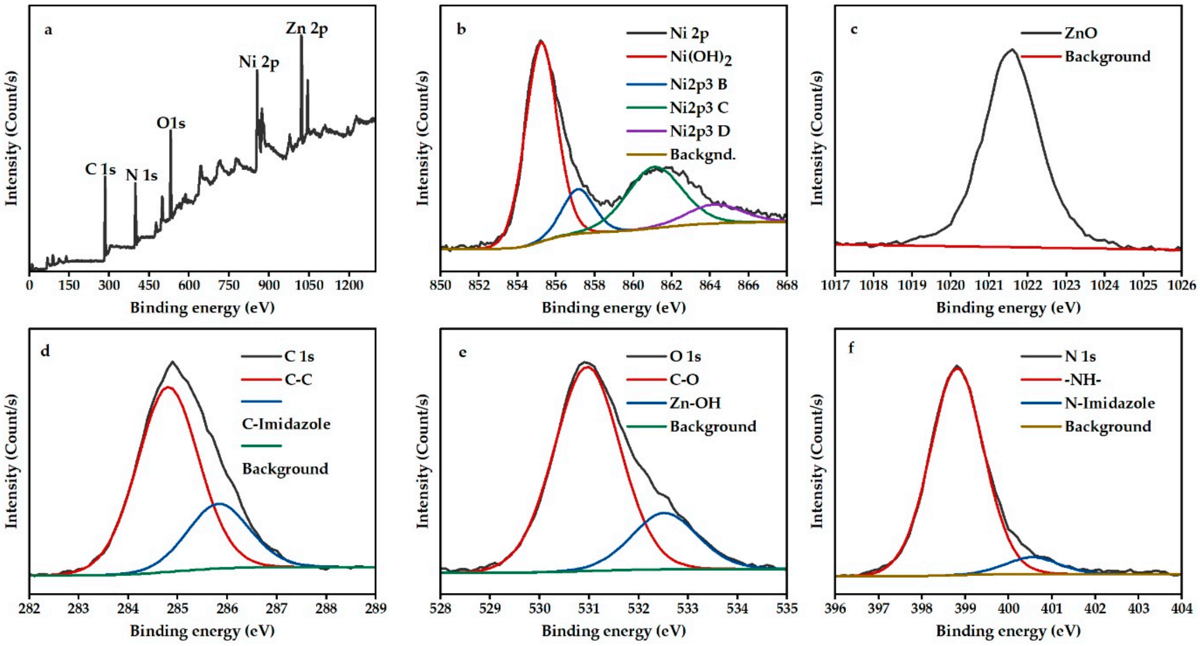


**Figure S1.** Surface elemental binding energies & distributions: (a) XPS survey spectrum of ZIF-8/NF, high-resolution and energy spectra of ZIF-8/NF (b) Ni 2p, (c) Zn 2p, (d) C 1s, (e) O 1s and (f) N 1s regions.

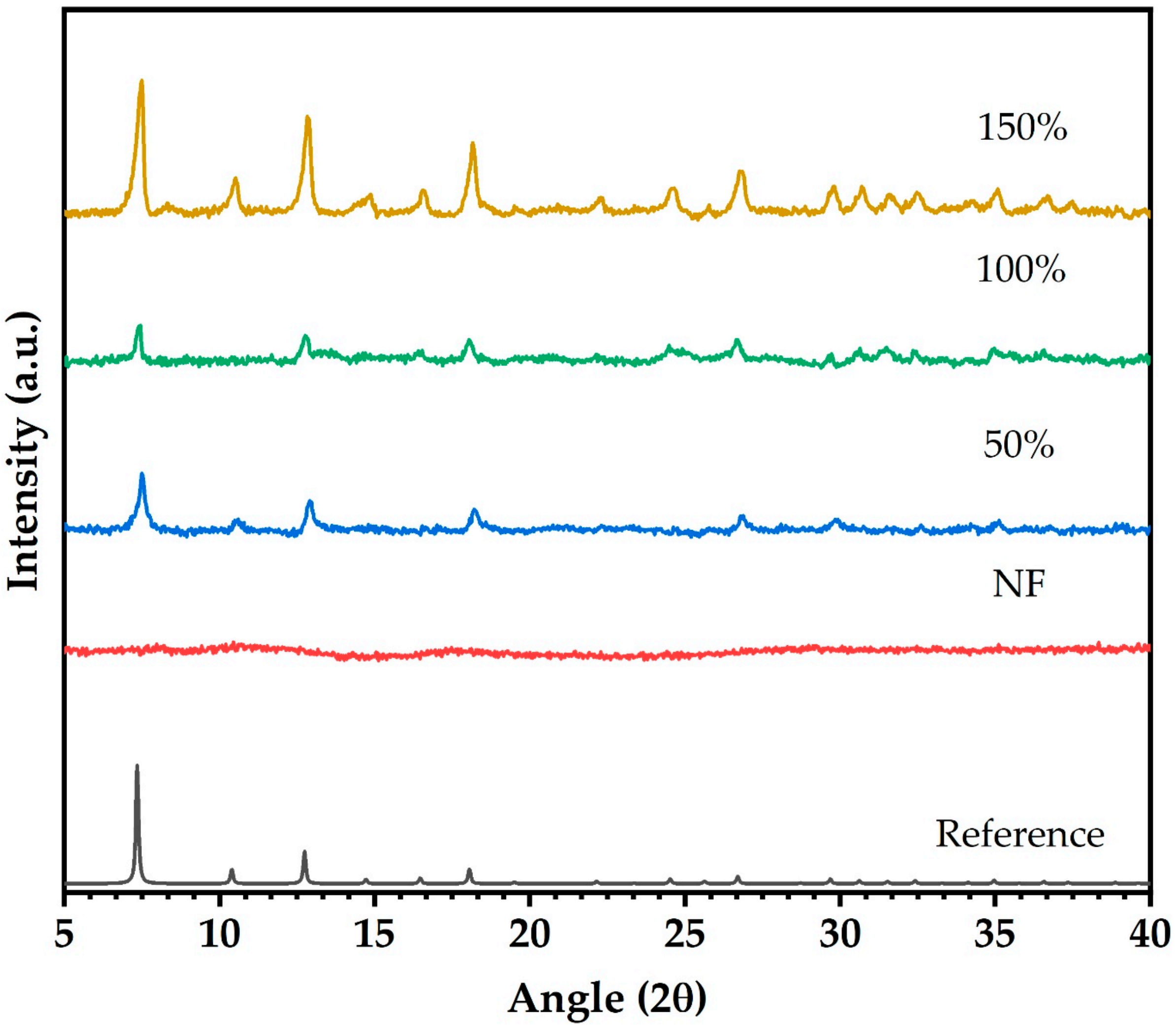


**Figure S2.** XRD patterns of ZIF-8/NF using different weight percent with identical mole ratio.

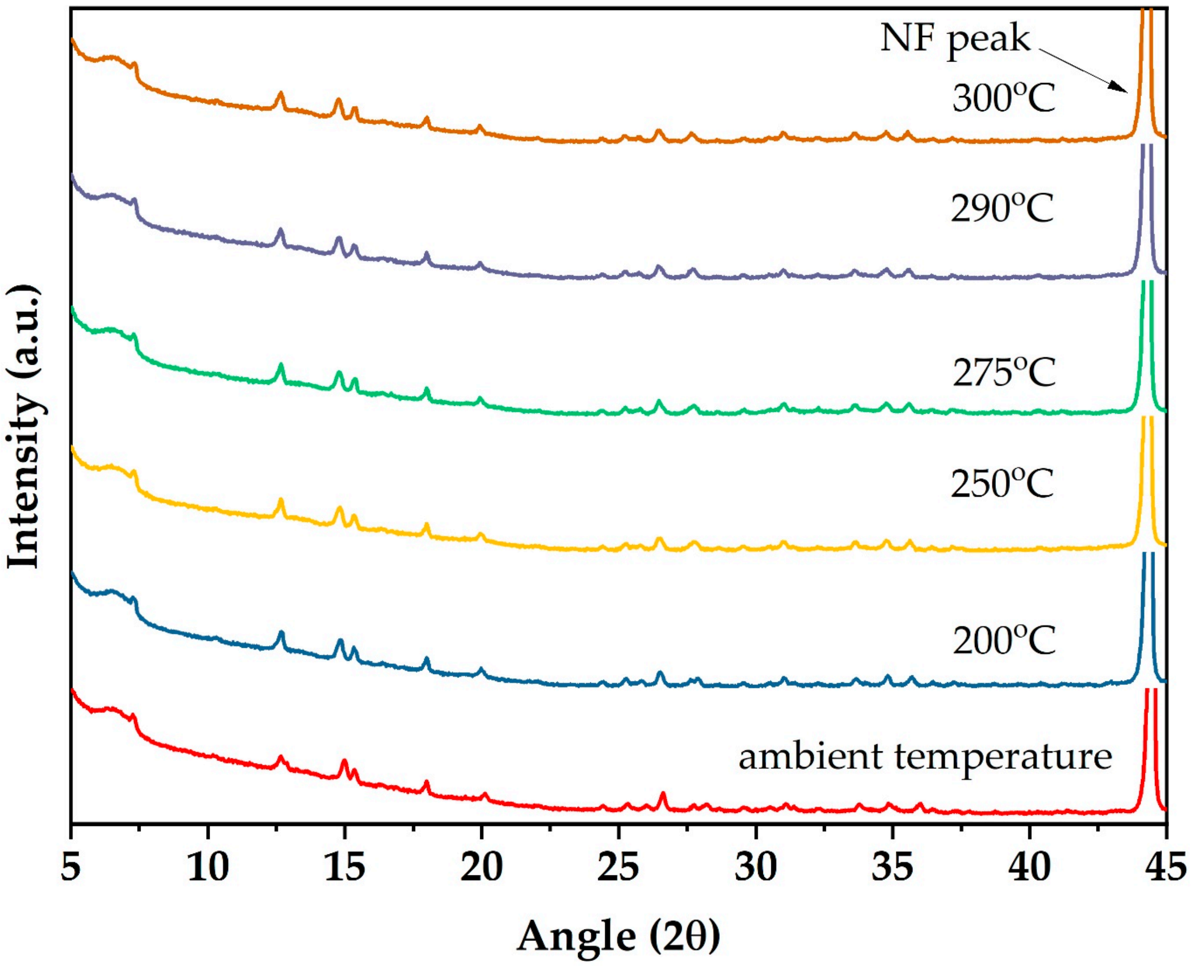

**Figure S3.** Temperature dependent study of ZIF-8/NF using non-ambient XRD technique.

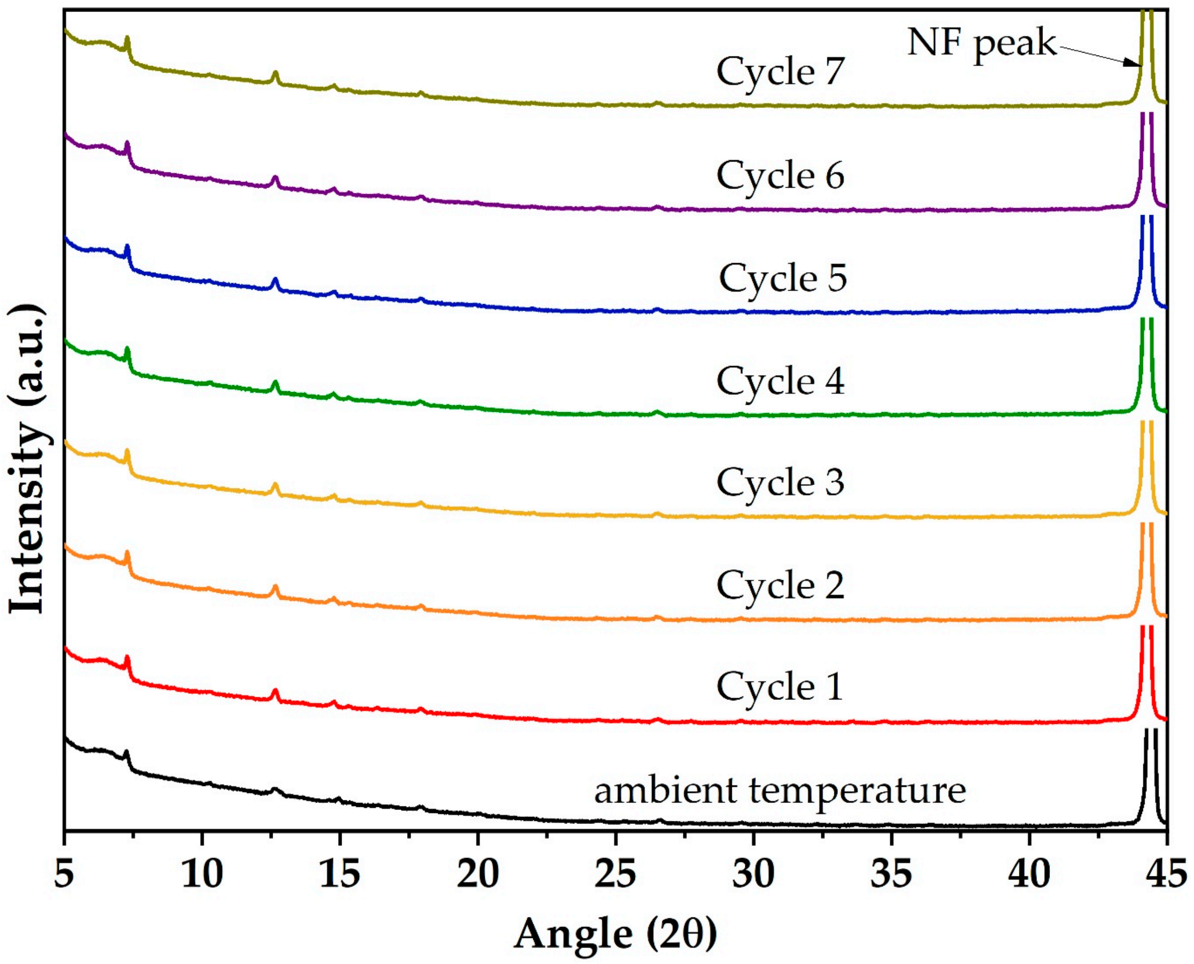


**Figure S4.** Non-ambient XRD patterns of ZIF-8/NF at 280°C for 7 repeated cycles.

**Table S1. The calibration using TD-GC/MS.**

| Compound | Tenax-TA | | | | ZIF-8/NF | | | |
|---|---|---|---|---|---|---|---|---|
| | Calibration range (ng/L) | Calibration equation | $R^2$ | LOD (ng/L) | Calibration range (ng/L) | Calibration equation | $R^2$ | LOD (ng/L) |
| 1-octen-3-ol | 0.89 – 8.05 | $y = 5197x - 4161$ | 0.977 | 2.7 | 0.89 – 8.05 | y = 1955x – 713 | 0.980 | 4.5 |
| 1-octen-3-one | 0.85 – 7.66 | $y = 5042x - 3642$ | 0.992 | 2.6 | 0.85 – 7.66 | y = 1636x – 733 | 0.992 | 2.6 |
| 3-octanone | 0.87 – 7.84 | $y = 4256x - 1906$ | 0.990 | 2.6 | 0.87 – 6.10 | y = 4788x – 1410 | 0.994 | 2.6 |

**Table S2. List of common phytosanitary volatiles in grapes and wines.**

| Analytes | Chemical Structure | Vapor pressure (mmHg) at 25°C |
|---|---|---|
| **(E)-2-Hexenal** | 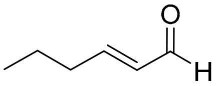 | 4.60E+00 |
| **3-Octanone** | 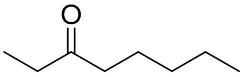 | 2.00E+00 |
| **1-Octen-3-one** | 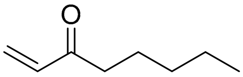 | 1.10E+00 |
| **Guaiacol** | 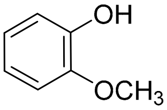 | 7.60E-02 |
| **4-Ethylguaiacol** | 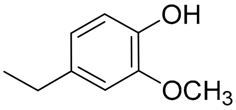 | 1.70E-02 |

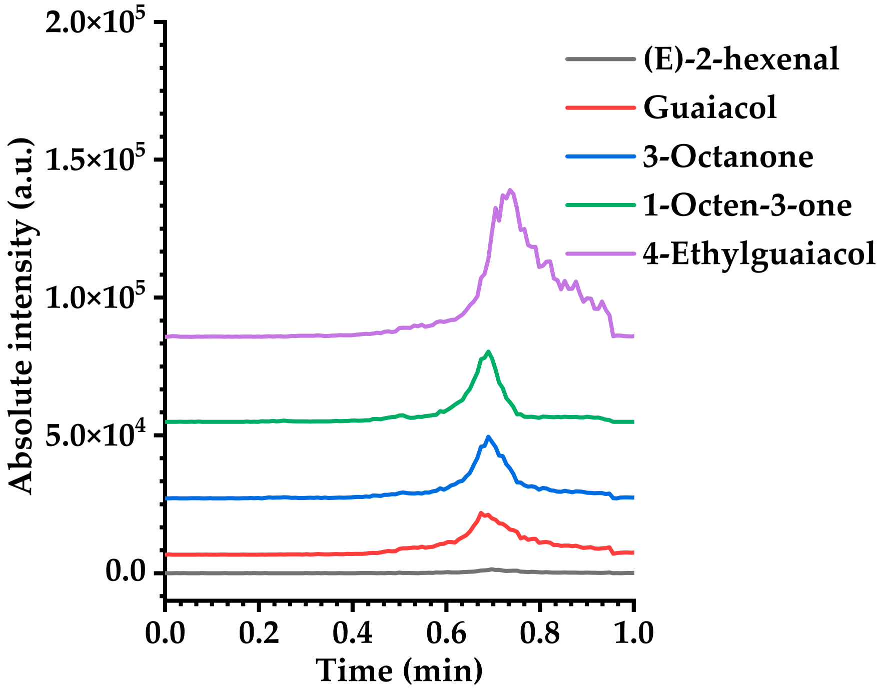


**Figure S5.** The extracted chromatogram at 280°C desorption temperature using ZIF-8/NF.

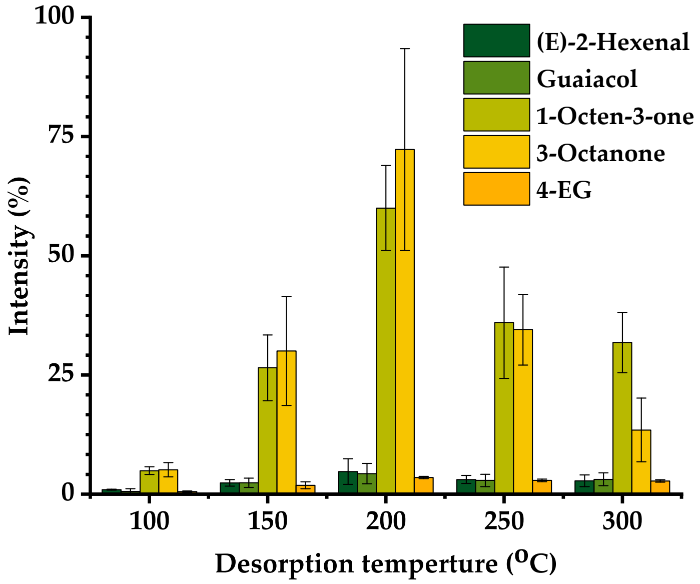


**Figure S6.** Desorption temperature dependent study of selected analytes using

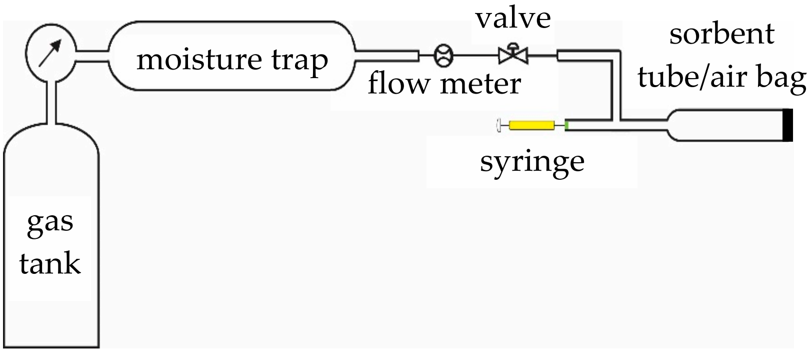


**Figure S7.** Schematic diagram for gas sampling.

The customized gas sample set up has thermal block with a digital temperature controller. The thermal block fits with Swagelok® Tube Fitting, union tee, ¼″ tube OD. One end is capped with stainless steel plug for ¼″ and 11-mm high temperature resistant GC inlet septa (Agilent, Australia). This part served as the sample injector/inlet. The second end is connected to a high purity nitrogen gas that passes through from moisture trap and digital airflow controller. The third end is the volatile outlet which can be connected to sample gas bag or thermal desorption tube.
